# Molecular Surveillance of MRSA in Raw Milk Provides Insight into MRSA Cross Species Evolution

**DOI:** 10.1128/spectrum.00311-23

**Published:** 2023-06-01

**Authors:** Zhenbiao Zhang, Jianzhong Wang, Hejia Wang, Liu Zhang, Wei Shang, Zekun Li, Li Song, Ting Li, Min Cheng, Chunping Zhang, Qi Zhao, Shan Shen, Mingquan Cui

**Affiliations:** a Shanxi Key Laboratory for Modernization of TCVM, College of Veterinary Medicine, Shanxi Agricultural University, Jinzhong, Shanxi, People’s Republic of China; b China Institute of Veterinary Drug Control, Beijing, People’s Republic of China; c Key Laboratory of Animal Antimicrobial Resistance Surveillance, Ministry of Agriculture and Rural Affairs, People’s Republic of China; d College of Agricultural Standardization, Shanxi Agricultural University, Jinzhong, Shanxi, People’s Republic of China; e No.988 Hospital of Joint Logistic Support Force, Zhengzhou, Henan, People’s Republic of China; f Shandong Provincial Hospital (Group) Ludong Hospital, Yantai, Shandong, People’s Republic of China; University of Calgary

**Keywords:** methicillin-resistant *Staphylococcus aureus*, milk, foodborne, multidrug-resistant, bioinformatic analysis

## Abstract

Methicillin-resistant Staphylococcus aureus (MRSA) in foods has been associated with severe infections in humans and animals worldwide. In the present study, the molecular characteristics of livestock-associated MRSA (LA-MRSA) and human-associated MRSA (hMRSA) isolates obtained in China, as well as MRSA isolates obtained from raw milk in 2018, were investigated. In total, 343 (20.38%; 343/1,683) S. aureus isolates were obtained from 1,683 raw milk samples from 100 dairy farms in 11 provinces across China. Among these, 49 (2.91%; 49/1,683) were *mecA*-positive MRSA. All LA-MRSA isolates were resistant to penicillin and highly resistant to erythromycin, sulfisoxazole, and clindamycin. Bioinformatic analysis the 49 genomes of LA-MRSA and 71 previously published hMRSA genomes isolated from Chinese individuals in 2018 indicated that *blaZ*, *erm*, *ant(6)-Ia*, *aph(3′)-III*, *tet*(K), *cat,* and *aph(2″)-Ia* were more prevalent in MRSA from raw milk (*P < *0.05) compared to hMRSA. Additionally, hMRSA isolates were more significantly associated with ST5 (*P < *0.01) compared to LA-MRSA; in contrast, ST338 was more prevalent among LA-MRSA isolates (*P < *0.01). Likewise, the SCC*mec* type II was only detected in hMRSA isolates, whereas SCC*mec* type V and IV were more prevalent among LA-MRSA (*P < *0.01). Furthermore, core-genome phylogenetic analysis showed the endemic characteristics of LA-MRSA in local provinces, as well as the close evolutionary relationships between MRSA from cattle and humans. Finally, homology analysis of *mecA* and *blaZ* genetic contexts revealed a high possibility of horizontal transmission of MRSA resistance genes among raw milk-associated and hMRSA strains, which increases the risk for public health.

**IMPORTANCE** Methicillin-resistant Staphylococcus aureus (MRSA) is considered a public health concern as it is resistant to multiple antibiotics, thus being in zoonotic transmission of antibiotic resistance genes. MRSA causes serious public health issues and leads to hard-to-treat infections in humans and animals; therefore, it was meaningful to determine the prevalence of MRSA in raw milk samples and investigate phenotype and genotype of antimicrobial resistance and molecular characteristics in livestock-associated MRSA (LA-MRSA) and human-associated MRSA (hMRSA) in China, which could provide a theoretical basis for preventing and controlling the spread of MRSA between livestock and humans.

## INTRODUCTION

Staphylococcus aureus is among the leading causes of foodborne associated illnesses, with raw milk and dairy products frequently associated with S. aureus contamination, thereby acting as a vehicle or source of S. aureus to humans (1). Food products associated with staphylococcal food poisoning (SFP) include, but are not limited to, milk and milk-based creams, butter, cheese, salads, deli products, and sandwich fillings, all of which usually contain raw milk (2). In addition, contamination by S. aureus decreases food quality and leads to substantial food loss, thus being considered a recognized pathogen involved in food poisoning outbreaks due to the existence of enterotoxigenic strains. Among SFP symptoms, the most common are gastroenteritis, diarrhea, and vomiting (3). In addition, considering food safety and farming economy, S. aureus is also a common pathogen causing mastitis in dairy livestock, which leads to poor animal welfare, reduced milk production, and significant economic losses (4, 5).

Methicillin-resistant S. aureus (MRSA) is considered a public health concern since it is resistant to multiple antibiotics, but the zoonotic risk remains to be fully explored (6). MRSA causes serious public health issues and leads to hard-to-treat infections in humans and animals, which include pneumonia, endocarditis, osteomyelitis, sepsis, or toxic shock syndrome, among others (7). Livestock-associated MRSA (LA-MRSA) is often highly resistant to β-lactam antibiotics, most commonly due to the presence of the *blaZ* gene and/or the *mecA/B/C* genes (8). The *blaZ* gene encodes a serine beta-lactamase (BlaZ) that forms the same type of acylase intermediate as the transpeptidase of penicillin-binding protein 2 (PBP2), and BlaZ itself is a lipoprotein whose expression is inducible and controlled by *blaI* repressors and *blaR* sensors (8). BlaZ efficiently hydrolyzes these penicillins, and thus protects the PBPs of MSSA from inactivation (9). The *mecA* gene is expressed and regulated by *mecIR* in an MRSA prototype strain after drug induction, in which *mecIR* is homologous to *blaR* sensors (10). It has been shown that a high-level heterogeneous-to-homogeneous conversion of chromosomally encoded beta-lactamase resistance increased the transcription of *mecA* directly or indirectly, thereby increasing the levels of PBP2 (11). In addition, the *lsa*(E) gene conferring cross-resistance to pleuroclein, lincosamide, and streptogramin A (PLSA phenotype) was first identified in S. aureus, and is thought to be transferred from representatives of the genus *Enterococcus* (12). The *lsa*(E) gene is usually located in multidrug-resistant regions of chromosomal DNA, but also on plasmids (13), and has been increasingly reported in others bacterial species such as Erysipelothrix rhusiopathiae, Streptococcus suis, and Streptococcus agalactiae.

Whole-genome sequencing (WGS), multilocus sequence typing (MLST), *Staphylococcal* cassette chromosome *mec* (SCC*mec*) typing, and *spa* typing are sequencing-based molecular techniques generally used for studying the genetic background of MRSA isolates, the results of which increase the understanding of the transmission characteristics of MRSA. Additionally, different LA-MRSA lineages have evolved host-specific adaptions which promote their persistence in different hosts (14). Sequence type (ST) 398 is reportedly the most common lineage among LA-MRSA isolates worldwide (7), being known for its higher capacity to incorporate foreign DNA, and mainly harbors the SCC*mec* type IV or V (15). However, ST9 is the most prevalent clonal type among LA-MRSA isolated in China, which harbors four SCC*mec* types, i.e., III, IV, V, and XII, as well as multiple *spa* types (16). Moreover, more recent studies have confirmed that ST9-t899 is epidemic among pork-related MRSA isolates in China (17, 18), although their molecular characteristics of MRSA isolates obtained from raw milk of health dairy cows are unknown.

Therefore, the aims of the present study were: (i) to determine the prevalence of MRSA in raw milk samples in China in 2018, (ii) to investigate phenotype and genotype of antimicrobial resistance and virulence factors in LA-MRSA and hMRSA, and (iii) to determine the molecular characteristics of these MRSA isolates. Finally, we provide a theoretical basis for preventing and controlling the spread of MRSA between livestock and humans.

## RESULTS

### Prevalence and distribution of MRSA isolates.

**FIG 1:**
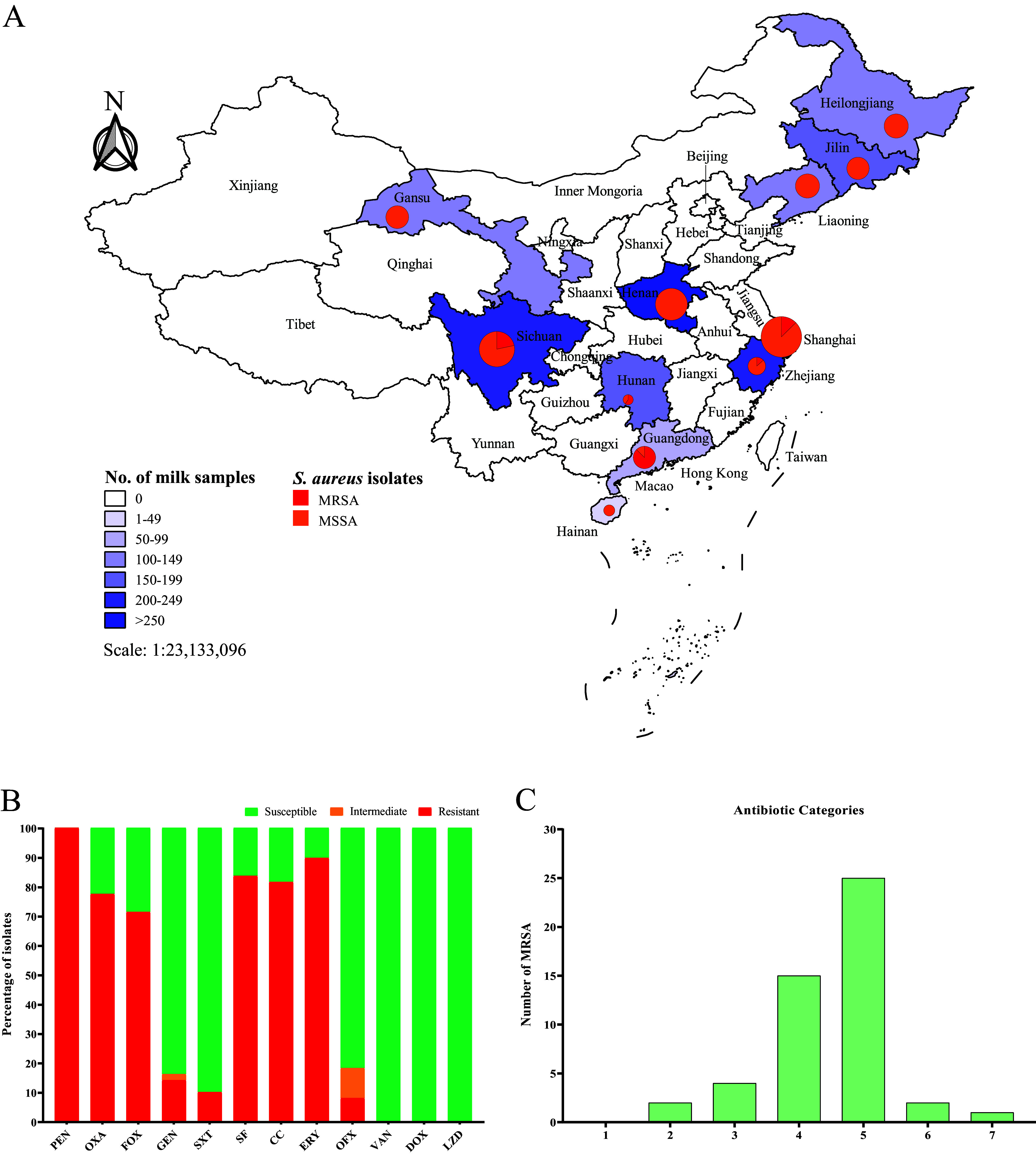
Map of the sampling locations and the prevalence of MRSA isolates, and the resistant profile of MRSA isolates from raw milk. (A) The 1,683 raw milk samples from 100 dairy farms were shown by blue color gradation stands in 11 different provinces of China. The S. aureus isolates were demonstrated using isometric pie charts and numbers, and the percentages of MRSA were included. (B) The rate of susceptibility, intermediate, and resistance to different antibiotics of cattle-MRSA isolates. PEN, penicillin; OXA, oxacillin; FOX, cefoxitin; GEN, gentamicin; SXT, trimethoprim-sulfamethoxazole; SF, sulfisoxazole; CC, clindamycin; ERY, erythromycin; OFX, ofloxacin; VAN, vancomycin; DOX, doxycycline; LZD, linezolid. (C) The number of multidrug-resistant cattle-MRSA isolates.

In total, 1,683 raw milk samples were collected from 100 dairy farms in 11 provinces in China. From these samples, 343 S. aureus isolates (343/1,683; 20.38%; 95% CI = 18.48 to 22.39) were obtained with each positive isolate originating from a different raw milk sample (Table 1; Fig. 1A). Among these, 49 (49/1,683; 2.91%; 95% CI = 2.16 to 3.83) *mecA*-positive MRSA isolates were identified, thus the prevalence of MRSA among S. aureus was 14.29% (49/343; 95% CI = 10.76 to 18.44) (Table 1). Interestingly, MRSA was not detected in samples originated from Hainan, Jilin, Heilongjiang, Liaoning, Henan, and Gansu provinces. Notably, the highest isolation rate of S. aureus (79/150; 52.67%; 95% CI = 44.36 to 60.87) was found among raw milk samples originating from Shanghai, which was significantly higher than samples from the other provinces (*P < *0.05). In contrast, the highest isolation rate of MRSA (21/80; 26.25%; 95% CI = 17.04 to 37.29) in samples from Guangdong province, which was significantly higher than that in samples from the other provinces (*P < *0.05). Furthermore, the percentage of MRSA among S. aureus isolates (87.50%; 21/24; 95% CI = 67.64 to 97.34) from Guangdong was more significant than other provinces, except for Hunan (*P < *0.05) (Table 1).

**TABLE 1:** The prevalence and distribution of MRSA from milk samples

The resource of MRSA isolates	The no. of dairy farms	The no. of milk samples	S. aureus isolates (%; 95% CI)	MRSA isolates (%; 95% CI)	Percentage of MRSA among S. aureus (%; 95% CI)
Guangdong	1	80	24 (30.00; 20.26 to 41.28)	21 (26.25; 17.04 to 37.29)	87.50; 67.64 to 97.34
Hainan	1	25	6 (24.00; 9.36 to 45.13)	0	0
Jilin	6	180	24 (13.33; 8.73 to 19.19)	0	0
Heilongjiang	7	100	28 (28.00; 19.48 to 37.87)	0	0
Liaoning	2	100	28 (28.00; 19.48 to 37.87)	0	0
Hunan	2	150	5 (3.33; 1.09 to 7.61)	3 (2.00; 0.41 to 5.73)	60.00; 14.66 to 94.73
Zhejiang	1	224	15 (6.70; 3.80 to 10.80)	2 (0.89; 0.11 to 3.19)	13.33; 1.66 to 40.46
Sichuan	10	249	60 (24.10; 18.92 to 29.90)	13 (5.22; 2.81 to 8.76)	21.67; 12.07 to 34.20
Henan	6	325	49 (15.08; 11.37 to 19.44)	0	0
Gansu	1	100	25 (25.00; 16.88 to 34.66)	0	0
Shanghai	63	150	79 (52.67; 44.36 to 60.87)	10 (6.67; 3.24 to 11.92)	12.66; 6.24 to 22.05
Total	100	1,683	343 (20.38; 18.48 to 22.39)	49 (2.91; 2.16 to 3.83)	14.29; 10.76 to 18.44

### Antimicrobial susceptibility profile of MRSA isolates.

All LA-MRSA isolates were resistant to penicillin (100%; 49/49); in addition, LA-MRSA isolates were highly resistant to erythromycin (89.80%; 44/49), sulfisoxazole (85.71%; 42/49), and clindamycin (81.63%, 40/49). Conversely, MRSA isolates showed low resistance to gentamicin (14.29%; 7/49), trimethoprim-sulfamethoxazole (10.20%; 5/49), and ofloxacin (6.12%; 3/49) (Fig. 1B; Table 2). All LA-MRSA isolates were susceptible to vancomycin, doxycycline, and linezolid (Fig. 1B; Table S2). Moreover, 47 out of 49 LA-MRSA isolates were resistant to three or more categories of antibiotics, thereby MDR-MRSA prevalence was 95.92% (Fig. 1C). Notably, LA-MRSA strain SH81 was resistant to seven classes of antimicrobial agents (Fig. 1C; Table S2). In particular, MIC_50_ and MIC_90_ of erythromycin, sulfisoxazole, and clindamycin for LA-MRSA isolates reached the highest test concentrations, i.e., 256 μg/mL, 512 μg/mL, and 128 μg/mL, respectively (Table 2).

**TABLE 2:** MIC (μg/mL) of antimicrobial agents for MRSA isolates from milk samples

Antimicrobial agents	Abbreviations	Test range (μg/mL)	MIC breakpoints (μg/mL)			MRSA resistance (%; 95% CI)	MIC_50_	MIC_90_
			S	I	R^*a*^			
Penicillin	PEN	0.125 to 256	≤ 0.125	—	≥ 0.25	49 (100.00; 92.75 to 100.00)	16	32
Oxacillin	OXA	0.125 to 256	≤ 2	—	≥ 4	38 (77.56; 63.38 to 88.22)	4	4
Cefoxitin	FOX	0.5 to 256	≤ 4	—	≥ 8	35 (71.43; 56.74 to 83.42)	16	32
Gentamicin	GEN	0.125 to 256	≤ 4	8	≥ 16	7 (14.29; 5.94 to 27.24)	1	32
Trimethoprim-sulfamethoxazole	SXT	0.125/2.375 to 32/608	≤ 2/38	—	≥ 4/76	5 (10.20; 3.40 to 22.23)	0.125/2.375	1/19
Sulfisoxazole	SF	16 to 512	≤ 256	—	≥ 512	42 (85.71; 72.76 to 94.06)	512	512
Clindamycin	CC	0.03 to 128	≤ 0.5	1 to 2	≥ 4	40 (81.63; 67.98 to 91.24)	128	128
Erythromycin	ERY	0.125 to 256	≤ 0.5	1 to 4	≥ 8	44 (89.80; 77.77 to 96.60)	256	256
Ofloxacin	OFX	0.125 to 256	≤ 1	2	≥ 4	3 (6.12; 1.28 to 16.87)	1	2
Vancomycin	VAN	0.25 to 128	≤ 2	4 to 8	≥ 16	0	1	1
Doxycycline	DOX	0.125 to 256	≤ 4	8	≥ 16	0	2	2
Linezolid	LZD	0.5 to 128	≤ 4	—	≥ 8	0	2	2

a R, resistant; I, intermediate; S, susceptible; “—” represents no data.

### Prevalence of antibiotic resistance genes and virulence genes.

**FIG 2:**
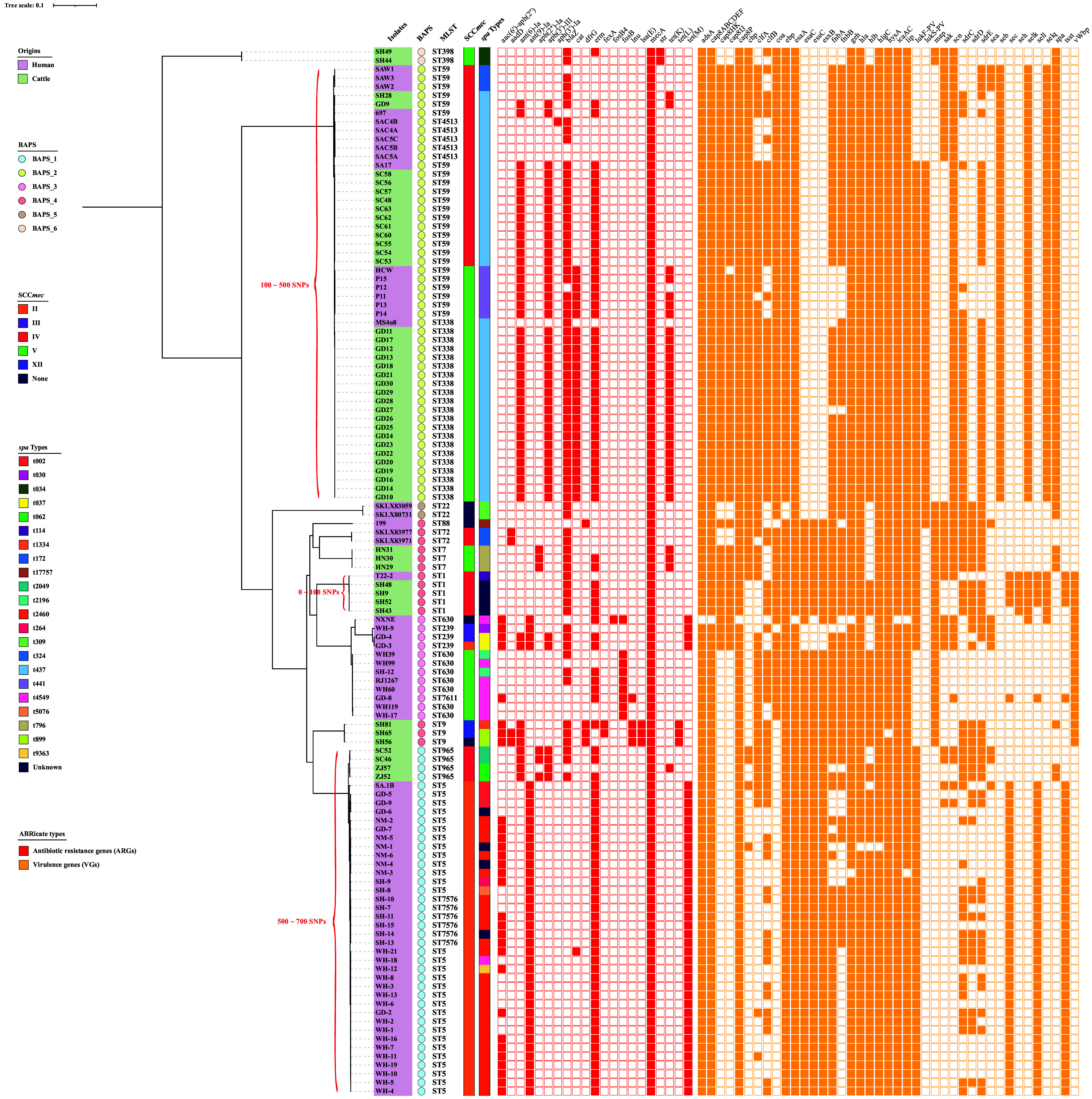
The core-genome phylogenetic tree and molecular characteristics of MRSA. Containing origins, phylogroups, MLST, SCC*mec* types, *spa* types, antibiotic resistance genes, and virulence genes of isolates from cattle and humans. The origins, phylogroups, SCC*mec* types, and *spa* types are differentiated by different colors. Antibiotic resistance genes and virulence genes are denoted by filled squares for the presence and empty squares for the absence.

All LA-MRSA and hMRSA isolates harbored the *mecA* gene (100%; 120/120). Moreover, *erm* (78.33%; 94/120) was the most prevalent resistance gene in MRSA; the variants *erm(A)* (*n* = 38), *erm(B)* (*n* = 45), and *erm(C)* (*n* = 19) were also detected. The prevalence of *blaZ* (97.96%; 48/49), *erm* (87.76%; 43/49), *ant(6)-Ia* (79.59%; 39/49), *aph(3′)-III* (73.47%; 36/49), *tet*(K) (53.06%; 26/49), *cat* (40.82%; 20/49), and *aph(2″)-Ia* (12.24%; 6/49) was significantly higher among raw milk MRSA isolates (*P < *0.05) compared to that in hMRSA genomes (Fig. 2). Conversely, the prevalence of *ant(9)-Ia* (56.34%; 40/71), *tet*(M) (56.34%; 40/71), and *aac(6′)-aph(2″)* (38.03%; 27/71) among human isolates was significantly higher than that among cattle-associated MRSA isolates (*P < *0.05) (Fig. 2). Interestingly, *aph(2″)-Ia*, *lsa*(E), *tet*(L), *fexA*, and *str* were only found in raw milk MRSA isolates, although *ant(9)-Ia*, *tet*(M), *fusB*, *aph(3′)-Ia*, and *fosB4* were just absent among hMRSA isolates.

Considering virulence genes (VGs), all MRSA isolates carried *aur*, *cap8GLMNO*, *esaB*, *essAB*, *esxA*, *hld*, *hlgAB*, *icaBDR*, *isdABCDEFG*, *sbi*, *srtB*, and *sspABC*, as revealed by ABRicate. Among VGs, the prevalence of *fnbA*, *sdrCE*, *scn*, *coa*, *fnbB*, *seb*, *clfAB*, *chp*, *selkq*, *cap8HKIJ*, and *lukS-PV* was significantly higher among MRSA isolates in health dairy cows compared to hMRSA isolates (*P < *0.05) (Fig. 2). In contrast, the prevalence of *essC*, *esaC*, *esxB*, *sec*, *sell*, and *tsst* among hMRSA isolates was more remarkable than among cattle MRSA isolates (*P < *0.05) (Fig. 2). The prevalence of *lukF-PV* and *lukS-PV* in LA-MRSA was 85.71% (42/49) and 63.27% (31/49), whereas a lower rate was found among hMRSA isolates, i.e., 76.06% (54/71) and 15.49% (11/71), respectively. Moreover, the enterotoxin gene *sel* (75.51%; 37/49) was found mostly in raw milk samples, followed by *seb* (67.35%; 33/49); whereas *sec* (53.52%; 38/71) and *sel* (26.76%; 19/71) were more common in hMRSA isolates.

### Phylogenetic and molecular analysis.

**FIG 3:**
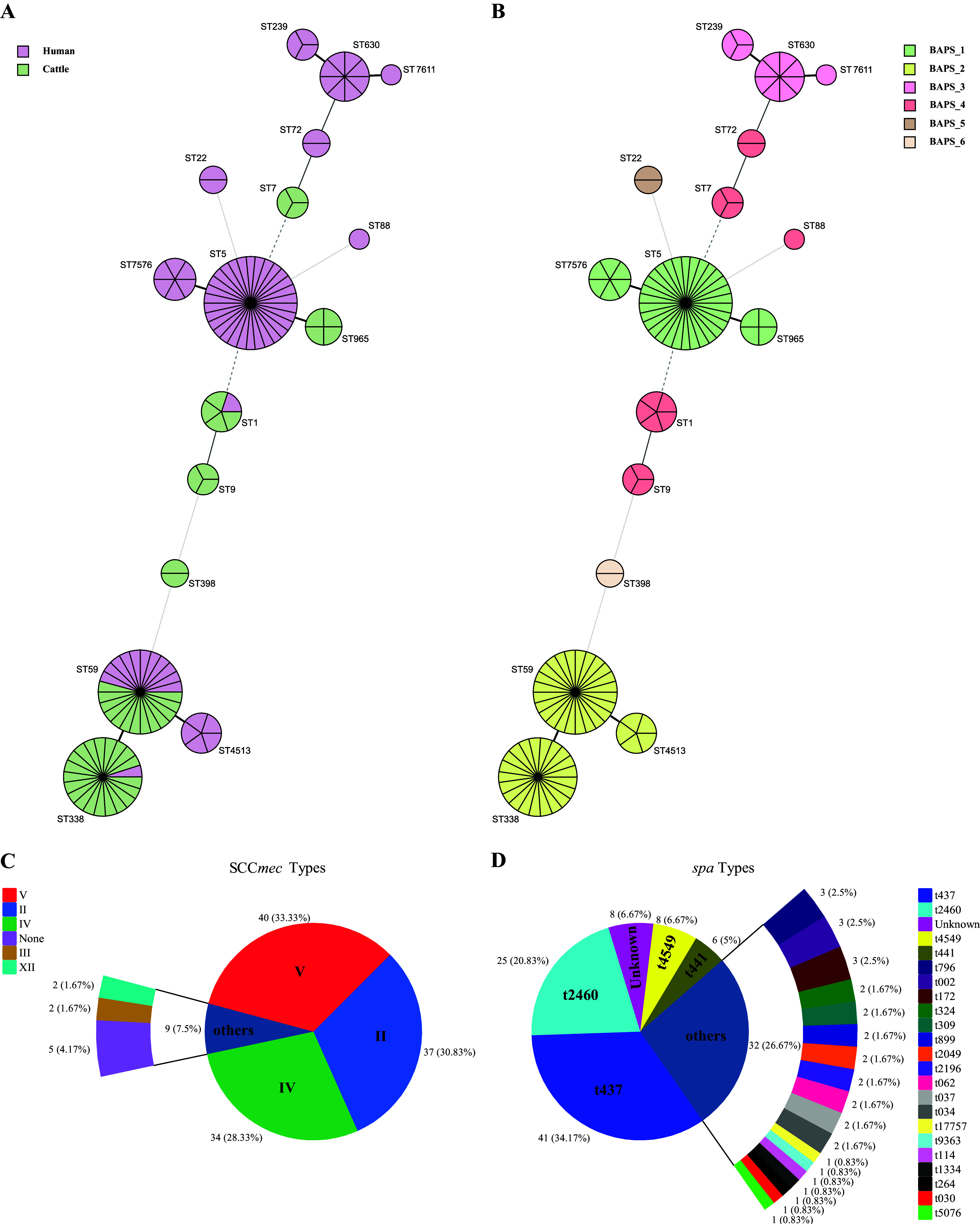
Minimum spanning trees of MLST typing, and SCC*mec* types and *spa* types. (A) STs were colored based on the different origins of cattle and humans. (B) STs were colored based on different Bayesian population structures. The distribution of SCC*mec* types (C) and *spa* types (D) of MRSA isolates from cattle and human samples.

Minimum spanning tree analysis revealed that ST5 (42.25%; 30/71) was the dominant ST among hMRSA isolates, which was significantly higher (*P < *0.01) compared to cattle MRSA isolates. However, ST338 (40.82%; 20/49) was the primary ST in raw milk samples, which was significantly more prevalent (*P < *0.01) than in hMRSA isolates (Fig. 2; Fig. 3A). Core-genome phylogenetic analysis showed that 70,261 SNPs were present among all MRSA strains. In contrast, SNPs difference among LA-MRSA isolates from the same provinces was smaller than that in isolates from different provinces, thus indicating the endemic characteristics of LA-MRSA. In addition, hMRSA strains MS4o8, SA17, and T22-2 were compared with the partial genomes of MRSA isolates obtained from raw milk samples from Guangdong, Sichuan, and Shanghai with SNP difference < 100, respectively (Fig. 2). Moreover, hierBAPS analysis revealed six MRSA lineages among LA-MRSA (*n* = 49) and hMRSA (*n* = 71) isolates. More specifically, BAPS_3 (*n* = 12) and BAPS_5 (*n* = 2) were exclusively found among hMRSA, whereas BAPS_6 (*n* = 2) was only found among LA-MRSA; the other lineages comprised MRSA isolates obtained from the two sources (Fig. 2; Fig. 3B). Collectively, the above results suggested a possibility of horizontal transmission of different ST types in MRSA isolates between foodborne and human-associated MRSA strains.

Furthermore, SCC*mec* type V (33.33%; 40/120), II (30.83%; 37/120), and IV (28.33%; 34/120) were dominant among LA-MRSA and hMRSA (Fig. 3C); however, SCC*mec* type II was exclusively detected among hMRSA strains (52.11%; 37/71). The prevalence of SCC*mec* type V and IV was significantly higher (*P < *0.01) among MRSA from raw milk isolates, i.e., 51.02% (25/49) and 42.86% (21/49), in comparison to human isolates, 21.13% (15/71) and 18.31% (13/71), respectively (Fig. 2; Fig. 3C). Interestingly, ST338 (*n* = 20) was the most predominant ST among SCC*mec* type V LA-MRSA isolates, whereas ST360 (*n* = 7) and ST59 (*n* = 6) predominated among hMRSA isolates. In addition, SCC*mec* type II was strongly associated with ST5 (*n* = 30) and its variant ST7576 (*n* = 6). In contrast, SCC*mec* type IV was often associated with ST59 (*n* = 18) and its variant ST4513 (*n* = 5), which was distributed among isolates from different sources. Moreover, SCC*mec* type IV-ST9 (*n* = 2) and type III-ST239 (*n* = 2) were found among isolates, and four isolates could not be SCC*mec* typed (Fig. 3C; Fig. S1A).

Considering *spa* typing, 22 *spa* types were found among MRSA isolates, which were dominated by t437 (34.17%; 41/120) and t2460 (20.83%; 25/120) (Fig. 3D). t437 was found mostly among MRSA isolates from cattle (*n* = 33), which belonged to ST59 (*n* = 15) and its variants ST338 (*n* = 21), as well as to ST4513 (*n* = 5); in contrast, t2460 was found among human isolates, which belonged to ST5 (*n* = 20) and its variant ST7576 (*n* = 5) (Fig. 3D; Fig. S1B). In addition, t2460 (35.21%; 25/71) and t4549 (11.27%; 8/71) were particularly prevalent in hMRSA isolates, and whose prevalence was significantly higher compared to LA-MRSA from raw milk (*P < *0.05); however, t437 (67.35%; 33/49) of LA-MRSA was obviously more than that of hMRSA isolates (11.27%; 8/71) (*P < *0.01) (Fig. 2; Fig. 3D).

### Genetic context of marked antibiotic resistance genes in MRSA isolates.

**FIG 4:**
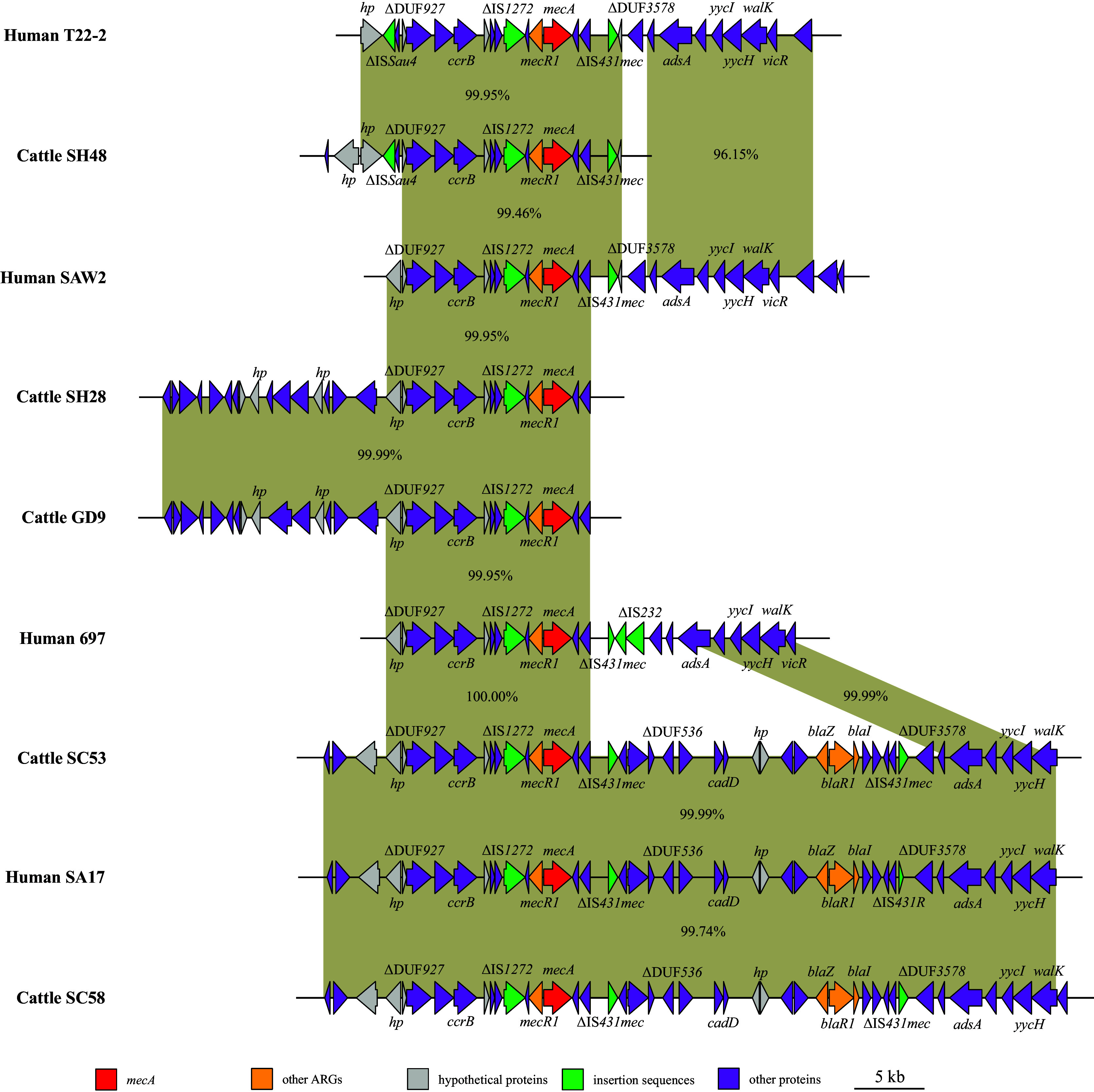
The homology and genetic environment of *mecA* gene. Characterization according to the SNPs difference < 500 between MRSA isolates from various origins of cattle and human in core-genome phylogenetic tree. The arrows indicate the directions of genes transcription, and different genes are represented by different colors. The region with ≥ 90.0% nucleotide sequence homology is gray. The Δ symbol represents the truncated genes.

Furthermore, contigs and gaps of MRSA isolates were identified by WGS analyses, upon which the backbone genome was assembled. The genetic context of marked antibiotic resistance genes (ARGs) *mec*A, *bla*Z, and *lsa*(E) was analyzed. First, SNPs difference < 500 was analyzed between MRSA isolates from cattle and human origins based on the core-genome phylogenetic tree (Fig. 2). Considering *mecA* characterization, glycerophosphoryl diester phosphodiesterase, MaoC family dehydratase, *mecA*, *mecR1*, type I restriction-modification system endonuclease, ΔIS*1272*, ΔDUF1643, ΔDUF960, hypothetical proteins (*hp*), *ccrB*, resolvase, and ΔDUF927 were encoded in common genetic skeleton structures (Fig. 4). It has been suggested that the presence of the insertion sequence ΔIS*1272* increased the diffusion risk of *mecA*, which partly explains the homology of the genetic context of *mecA* within the genus and with high similarly within different species. The genetic context of *mecA* in hMRSA strains HCW, MS4o8, and LA-MRSA strain GD11 was part of the structure of other strains, so it is not displayed.

**FIG 5:**
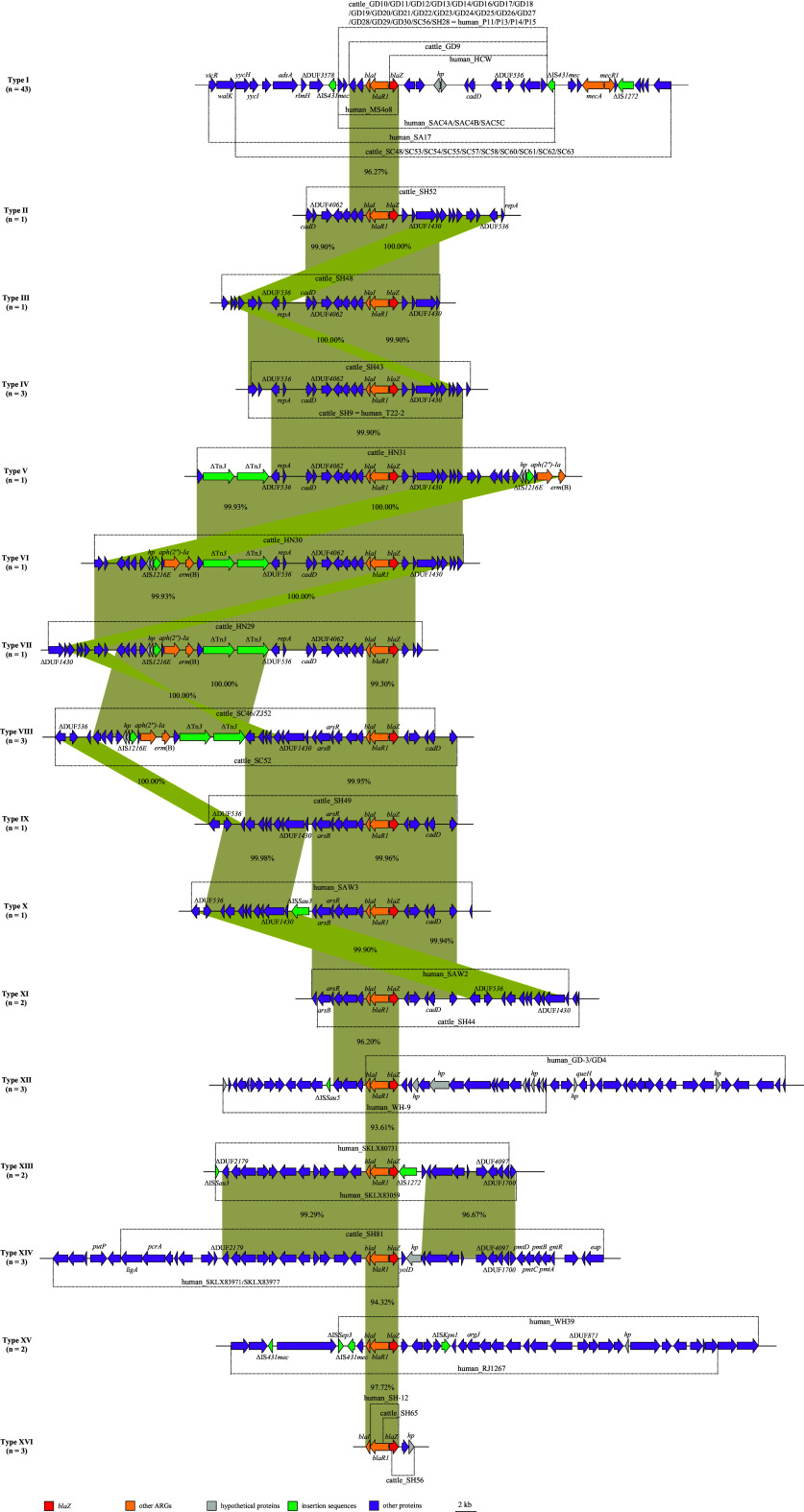
Genetic environment of *blaZ* in 120 MRSA isolates of cattle and humans. They were assigned to 16 different genetic environments and origins, and names of the strains were directly shown in the figure. Arrows indicate the directions of genes transcription, and different genes are represented by different colors. The region with ≥ 90.0% nucleotide sequence homology is gray or yellow. The Δ symbol represents the truncated genes. Isolates with different sizes of *blaZ* core area are represented by vertical lines.

Moreover, molecular analysis showed that *blaZ* (59.17%; 71/120) from cattle (97.96%; 48/49) and human (32.39%; 23/71) MRSA isolates was assigned to 16 different genetic contexts, of which all contained the structure *blaI*-*blaR1*-*blaZ* (Fig. 5). Type I (*n* = 43) was the most common genetic context, and incomplete insertion sequences ΔIS*431mec* and ΔIS*1272* were found both upstream and downstream *blaI-blaR1-blaZ*, respectively. Similarly, considering the genetic context of type V (*n* = 1), type VI (*n* = 1), type VII (*n* = 1), type VIII (*n* = 3), type X (*n* = 1), type XII (*n* = 3), type XIII (*n* = 2), and type XV (*n* = 2) contained transposon or insertion sequences, whose existence increased transfer efficiency of *blaZ* to varying degrees, and which were absent in other types (Fig. 5). The percentage sequence identity of *blaZ* gene was greater than 90%, demonstrating the horizontal transfer of *blaZ* between LA-MRSA and hMRSA isolates.

**FIG 6:**
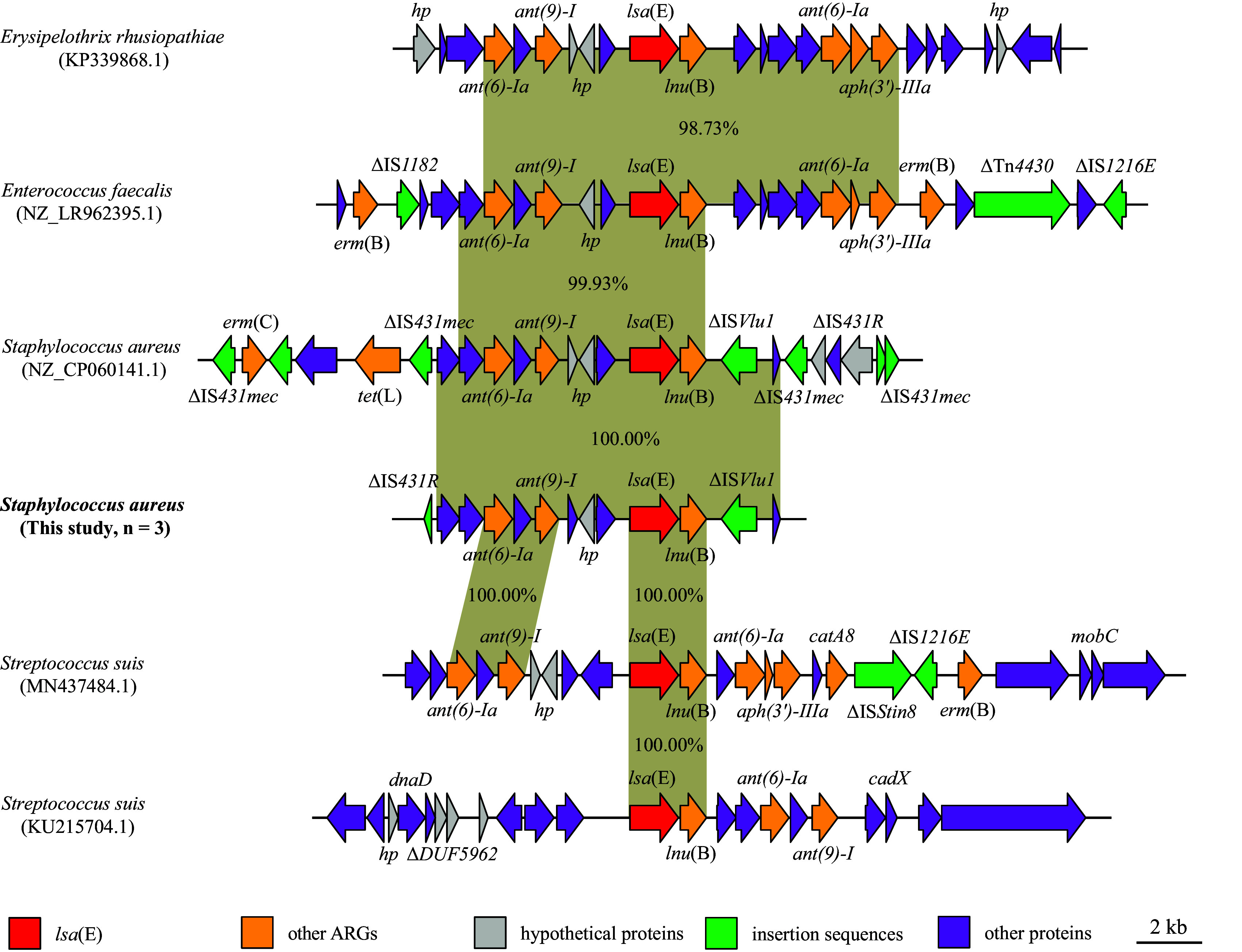
Comparison and homology analysis of *lsa*(E) gene-environment in different bacterial. The *lsa*(E) (*n* = 3) was only detected in ST9-cattle-MRSA and harbored one type of genetic environment in this study. The arrows indicate the directions of genes transcription, and different genes are represented by different colors. The region with ≥ 90.0% nucleotide sequence homology is gray. The Δ symbol represents the truncated genes.

Remarkably, *lsa*(E) (*n* = 3) was only detected in ST9-LA-MRSA within one type of genetic environment, which was bound to the lincosamide nucleotidyltransferase *lnu*(B) gene (Fig. 6). In the genetic context of *lsa*(E), the insertion sequences ΔIS*431R* and ΔIS*Vlu1*were found, respectively, upstream and downstream. Interestingly, the genetic backbone had 100% sequence percentage identity with S. aureus strain 2868B2 as available in the NCBI database (GenBank accession no. NZ_CP060141.1), as well as with *E. rhusiopathiae* strain Ery-11 (KP339868.1) and Enterococcus faecalis strain 28157_4#315 (NZ_LR962395.1); however, it differed from the genetic context of *lsa*(E) found in S. suis strain SC180 (MN437484.1) and S. suis strain nc286A7 (KU215704.1) (Fig. 6).

## DISCUSSION

S. aureus, a major pathogen that threatens public health, can acquire resistance to antibiotics and produce a series of virulence factors. The clinical usage of methicillin led to the emergence of MRSA, and this evolution continued as a result from human contact or food consumption, and MRSA has been increasingly found in companion and food animals (7). In the present study, raw milk samples from 100 dairy farms in 11 Chinese provinces were collected to investigate the prevalence and resistance of MRSA isolates obtained from cattle. In addition, the molecular characteristics of MRSA isolates from cattle origin were analyzed along with hMRSA isolates collected in China in 2018 available in the NCBI database. Moreover, 343 S. aureus isolates (20.38%; 343/1,683) were obtained from 1,683 raw milk samples. Interestingly, the isolation rate of S. aureus in the present study was significantly lower (*P < *0.01) than that related in previous studies conducted in the Philippines (41.94%, 39/93) (19) and Greece (29%, 162/559) (20). The isolation rate of S. aureus (52.67%; 79/150) in samples from Shanghai was similar to that from Shihezi (58.1%, 54/93) (21). Furthermore, 49 *mecA*-positive MRSA isolates (2.91%; 49/1,683) were confirmed, although no significant difference (*P > *0.05) was observed considering the findings reported in a study conducted in Italy (4%; 3/75) (22). Moreover, the percentage rate of MRSA recovery among S. aureus isolates in the present study was 14.29% (49/343), although no significant difference (*P > *0.05) was observed with the findings reported by Badua et al. (23.08%; 9/39), but was higher (*P < *0.01) than 0.99% (5/162) among milk samples from dairy goats in Greece (20).

Moreover, antimicrobial susceptibility profile of MRSA isolates was determined by broth microdilution method. MIC values indicated that MRSA isolates from cattle were resistant to penicillin (100%; 49/49) and highly resistant to erythromycin (89.80%, 44/49), sulfisoxazole (85.71%, 42/49), and clindamycin (81.63%, 40/49), which were similar to the findings reported in a previous study conducted in the Philippines (19), but was lower than the rates reported in a study conducted in Great Britain (23).

In contrast with the 71 published hMRSA genomes available on the NCBI database obtained from Chinese individuals in 2018, the prevalence of genes *blaZ* (97.96%; 48/49), *erm* (87.76%; 43/49), *ant(6)-Ia* (79.59%; 39/49), *aph(3′)-III* (73.47%; 36/49), *tet*(K) (53.06%; 26/49), *cat* (40.82%; 20/49), and *aph(2″)-Ia* (12.24%; 6/49) was higher in raw milk samples (*P < *0.05). In particular, the prevalence of *blaZ* and *tet*(K) in LA-MRSA isolates was also significant higher compared to findings in studies conducted in Southern Mozambique (24) and Xinjiang, China (21); however, the prevalence rate of *blaZ* and *aph(3′)-III* was similar to that reported in a study conducted in Northern Kenya with raw milk samples (25). In addition, all MRSA isolates carried VGs, including *aur*, *cap8GLMNO*, *esaB*, *essAB*, *esxA*, *hld*, *hlgAB*, *icaBDR*, *isdABCDEFG*, *sbi*, *srtB*, and *sspABC*. Moreover, the prevalence of *fnbA*, *sdrCE*, *scn*, *coa*, *fnbB*, *seb*, *clfAB*, *chp*, *selkq*, *cap8HKIJ*, and *lukS-PV* in raw milk was significantly higher than that found among hMRSA isolates (*P < *0.05). Moreover, the enterotoxin gene *seg* was the most frequently detected (41.9%), followed by *sei* (40.3%) and *sec* (6.5%) in a study conducted in Northern Portugal (26), which differed the prevalence rates for *sel* (46.67%, 56/120), *seb* (41.67%, 50/120), and *sec* (34.17%, 41/120) in the present study. Moreover, several studies showed that individuals who have close contact with livestock animals are at higher risk for colonization and infection with LA-MRSA, which carried multiple resistance genes, thus exacerbating uncontrollability such risks.

Genotyping of MRSA isolates was based on MLST, *SCCmec*, and *spa* typing. MLST, combined with SCC*mec* and *spa* typing, constitutes a useful tool for exploring the origin and evolution of S. aureus, and also provides a general naming system for S. aureus (27). ST239 and ST5 are hospital-associated MRSA (HA-MRSA) lineages that are prevalent in China and other Asian countries, whereas community-associated MRSA (CA-MRSA) lineages such as ST59, ST338, ST30, ST72, and ST8 are widespread in different geographical regions (7, 28). In the study, minimum spanning tree analysis confirmed that ST5 (42.25%; 30/71) was the dominant lineage among hMRSA isolates; however, the ST338 variant (40.82%; 20/49) was the predominant type in raw milk samples. Interestingly, ST59 coexisted in MRSA from human (*n* = 11) and cattle (*n* = 13) origin. In addition, Bayesian analysis showed that hMRSA and LA-MRSA isolates shared certain clusters in the core genome phylogenetic analysis. Combined with SNPs detection, the results indicated a high possibility of horizontal transmission of MRSA isolates could occur between raw milk- and human-derived strains, which increases the risks to public safety. Animal-to-human transmission can occur through three main routes, i.e., direct contact, environmental contamination, or handling of products from infected animals; however, MRSA-contaminated raw milk is one of the most direct and dangerous routes for oral transmission to humans.

In total, 13 SCC*mec* types were identified in cattle and human MRSA isolates. Among these, SCC*mec* type V (33.33%; 40/120), II (30.83%; 37/120), and IV (28.33%; 34/120) were predominant among MRSA isolates, whereas SCC*mec* type IV was the most predominant type and accounted for nearly half of all MRSA isolates reported in a previous study (19, 29). In contrast, STs within SCC*mec* types II, III, and IV were similar to those reported in another study (29). Among *spa* types, 22 *spa* types were identified in total, among which predominated t437 (34.17%; 41/120) and t2460 (20.83%; 25/120), which was similar to the findings reported in a study with HA-MRSA isolates which showed that *spa* types t437 and t2460 are highly epidemic in China.

Moreover, the analysis of the genetic context of *mecA* in MRSA isolates (difference in SNPs prevalence < 500) indicated that the homology of *mecA* was similar within the genus (either human or cattle origin) and highly similar within different species, thus demonstrating that LA-MRSA found in contaminated raw milk increases the diffusion risk of MRSA between veterinary and human spheres, which was consistent with the findings in a previous study with MRSA in pork (30). Moreover, molecular analysis revealed that *blaZ* was found within 16 different genetic contexts, all of which contained the structure *blaI*-*blaR1*-*blaZ*. In particular, type I (*n* = 43) was the most common genetic backbone, and incomplete insertion sequences ΔIS*431mec* and ΔIS*1272*were found both upstream and downstream *blaI-blaR1-blaZ*, respectively, which was similar to genetic contexts reported in previous studies conducted in Switzerland (31, 32). In addition, the percentage of sequence homology in the *blaZ* genetic context among different sources was greater than 90%, which further strengthens the hypothesis of horizontal transfer of *blaZ* between LA-MRSA and hMRSA isolates. In contrast, *lsa*(E) (*n* = 3) was detected only in ST9-LA-MRSA, commonly associated with *lnu*(B), and insertion sequences ΔIS*431R* and ΔIS*Vlu1* were found upstream and downstream the *lsa*(E)-*lnu*(B) structure. This genetic backbone had 100% sequence identity with the corresponding sequences of S. aureus strain 2868B2 available in the NCBI database (GenBank accession no. NZ_CP060141.1) (33). Thus, the information obtained from the characterization of marker genes and the genetic context can be used epidemiologically to elucidate outbreaks, identify possible sources of colonization (livestock or humans), and differentiate between community- and hospital-acquired strains. Moreover, it is important to identify and limit the emergence of MRSA strains belonging to the ST9-t3446-*SCCmec* type IX genotype and/or carrying acquired drug resistance genes *mecA*, *blaZ*, *aac (6′)-aph (2″)*, *aadD*, *ant (6)-Ia*, *lsa* (E), *dfrG*, *tet* (M), *fexA*, and *lnu* (B) as reported elsewhere (34).

Herein, a large-scale investigation of antimicrobial resistance in MRSA isolates obtained from raw milk samples from dairy farms across China was conducted, which enable comparing the molecular characteristics of LA-MRSA and hMRSA. All MRSA isolates evaluated in the present study were resistant to penicillin and highly resistant to erythromycin, sulfisoxazole, and clindamycin. Except for *mecA*, *erm* was the most prevalent resistance gene found in MRSA isolates from cattle and human origins. Moreover, the number of ARGs carried by LA-MRSA was greater than that carried by hMRSA isolates; in particular, *blaZ*, *erm*, *ant(6)-Ia*, *aph(3′)-III*, *tet*(K), *cat*, and *aph(2″)-Ia* were significantly more prevalent in raw milk isolates than in the genomes of MRSA of human origin. In addition, the enterotoxin gene *sel* was predominant in milk, whereas hMRSA isolates commonly harbored *sec*. Furthermore, ST5 and ST338 were the most prevalent lineages among MRSA isolates of human and cattle origins, respectively. Additionally, the relationship of MRSA strains origin raw milk samples from the same province was closer in the core-genome phylogenetic tree. With the homology analysis of marked genetic environments of *mecA* and *blaZ*, all the results demonstrated that there was a highly possibility of horizontal transmission of MRSA isolates between foodborne raw milk and human-derived strains, which increased the risk to public safety.

## MATERIALS AND METHODS

### Sample collection and selection of bacterial isolates.

A total of 1,683 raw milk samples were aseptically collected from healthy dairy cows of scalable farms located in different Chinese provinces. All samples (collected as triple duplicates) were stored momentarily in an insulated icebox and transported to the laboratory immediately after collection. Then, 1 mL of raw milk sample was mixed with 9 mL of 7.5% sodium chloride broth, and the mixture was incubated overnight at 37°C for enrichment. An aliquot of the bacterial suspension was then transferred uniformly onto CHROMagar S. aureus isolation medium (CHROMagar, Paris, France) followed by incubation for 24 h at 37°C. One colony was then selected and inoculated in 1 mL of brain heart infusion (BHI) (Land Bridge, Beijing, China) for incubation for 6 h at 37°C under shaking. The identification of selected colonies as S. aureus was conducted using a Vitek 2 Compact GP ID Card (bioMérieux, Marcy-l’Étoile, France).

### Molecular identification of MRSA isolates.

DNA of identified S. aureus isolates was extracted from pre-enrichment BHI using the TIANamp Bacteria DNA Kit (Tiangen, Beijing, China) according to the manufacturer’s instructions. *mecA*-positive MRSA isolates were confirmed using multiplex PCR (PCR) targeting the *16S rRNA*, *nuc* and *mecA* genes as previously described.(35) After confirmation, a total of 1.5 mL of MRSA positive pre-enriched samples were stored for subsequent processing.

### Determination of antimicrobial susceptibility.

Antimicrobial susceptibility of MRSA isolates was conducted using the broth microdilution method. Tested antibiotics included 12 agents belonging to 10 different classes, i.e., penicillins (penicillin and oxacillin), cephalosporins (cefoxitin), fluoroquinolones (ofloxacin), aminoglycosides (gentamicin), lincosamides (clindamycin), macrolides (erythromycin), glycopeptides (vancomycin), oxazolidinones (linezolid), tetracyclines (doxycycline), and folate pathway antagonists (trimethoprim-sulfamethoxazole and sulfisoxazole). MICs were interpreted according to Clinical and Laboratory Standards Institute (CLSI) standards M100 ED32:2022 (http://em100.edaptivedocs.net/dashboard.aspx); S. aureus ATCC 29213 was used in assays as the quality control strain. The bacterial isolate was considered multidrug-resistant (MDR) when it showed resistance to three or more classes of antimicrobial agents based on Standardized International Terminology (36).

### Whole-genome sequencing and bioinformatics analysis.

DNA of confirmed MRSA isolates was used as template for the construction of indexed DNA libraries, and whole-genome sequencing was conducted using the Illumina HiSeq 2500 system by Bionova Biotech Co., Ltd. (Beijing, China). Obtained raw data were assembled using SPAdes Genome Assembler (v3.9.0) (37), and assembled genome data were submitted to the NCBI database (PRJNA828118). To determine the relationships and differences among MRSA isolates from humans and cattle, a joint analysis was conducted, including additional previously published 71 MRSA genomes from human isolates (hMRSA) which were collected in China in 2018 (Table S1). In addition, ARGs and VGs were identified using the modules Resfinder (38) and VFDB (39), respectively, in ABRicate (v1.0.0, https://github.com/tseemann/abricate). MLST was conducted using the SRST2 toolkit (v0.2.0) (40). SCC*mec* and *spa* typing were conducted using SCC*mec*Finder v1.2 and *spa*Typer v1.0 in Center for Genomic Epidemiology (CGE) Services (https://cge.cbs.dtu.dk/services/). RAST (v2.0) (41) and Prokka (v1.14.5) (42) were used to annotate ARGs and VGs in combination with the NCBI database. The BURST algorithm of BioNumerics (v7.6.3, Applied Maths, Sint-Martens-Latem, Belgium) was used to generate the minimum spanning tree. Single nucleotide polymorphisms (SNPs) in the core genome were compared and analyzed by Harvest (v1.1.2) (43), whereas hierBAPS (https://github.com/chengl7/hierBAPS) (44) was used to analyze the Bayesian population structure. Finally, Interactive Tree Of Life (iTOL) v5 (45) was used to display the SNPs core genome evolutionary tree which was constructed using FastTree2 (v2.1.11) (46).

### Statistical analysis.

Statistical significance was determined using Chi-square (χ^2^) and Fisher’s exact tests in SPSS software (version 22; IBM Corporation, Armonk, NY, USA) or GraphPad Prism (version 8.4.3; GraphPad Software, San Diego, CA, USA). The level of significance was set at *P < *0.05.

### Declarations.

Ethics approval and consent to participate: Not applicable.

Consent for publication: Not applicable.

Competing interests: No potential conflict of interest was reported by the authors.

### Data availability.

The whole-genome sequencing data of LA-MRSA presented in this study can be found in online repositories https://www.ncbi.nlm.nih.gov/. The names of the repository/repositories and accession number was PRJNA828118. Metadata information for 71 hMRSA strains isolated from human in 2018 of publicly available on NCBI was displayed in Table S1.
