# Supplementary figures and images for: Molecular Surveillance of MRSA in Raw Milk Provides Insight into MRSA Cross Species Evolution
Source: Microbiol Spectr. 2023 Jun 1;11(4):e00311-23. doi: 10.1128/spectrum.00311-23 (PMC10433870; doi:10.1128/spectrum.00311-23)

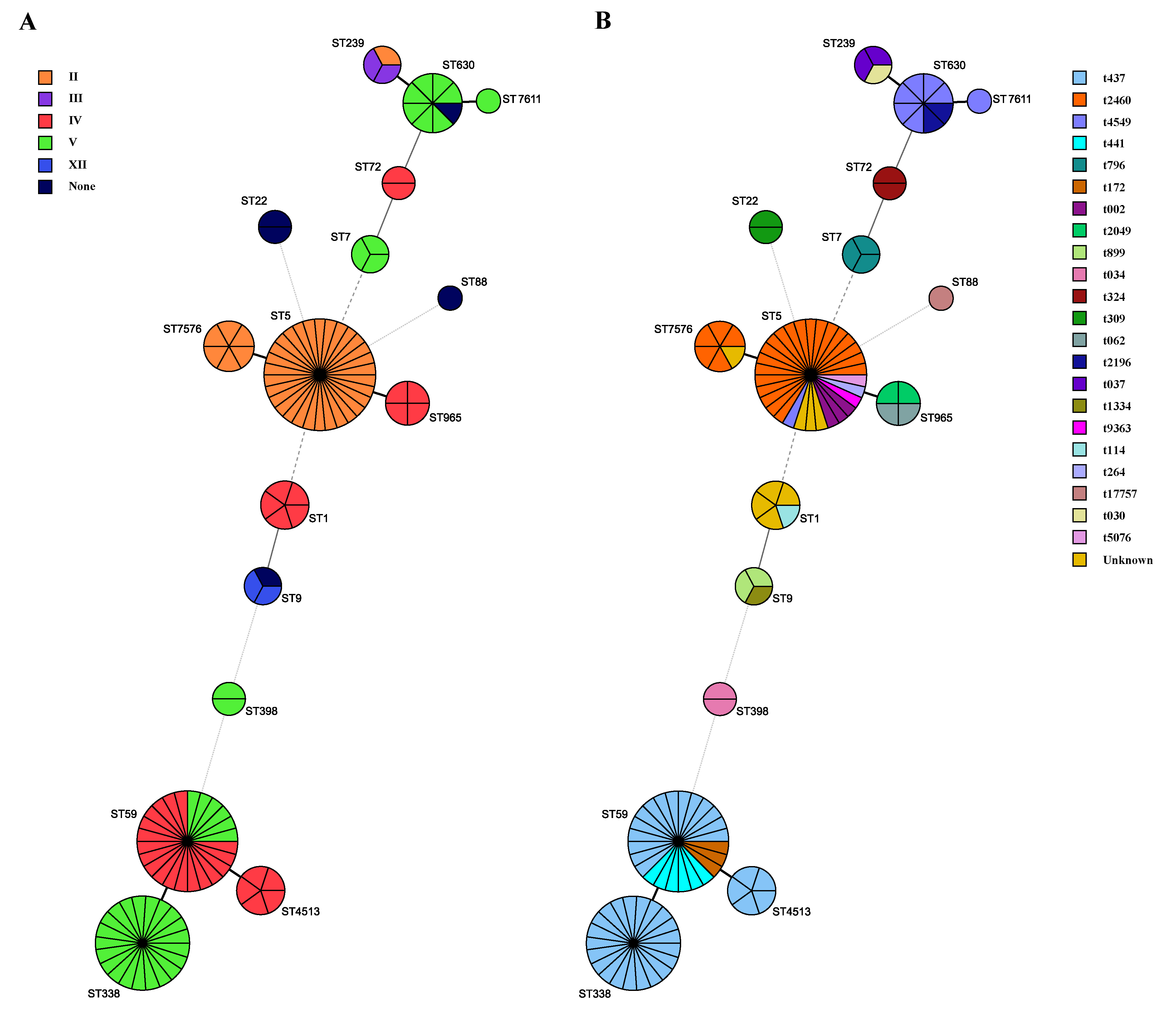

Supplement: Supplemental file 1 — Fig. S1. Download spectrum.00311-23-s0001.tif, TIF file, 1.5 MB [file spectrum.00311-23-s0001.tif]
