# Supplementary material for: Molecular Surveillance of MRSA in Raw Milk Provides Insight into MRSA Cross Species Evolution
Source: Microbiol Spectr. 2023 Jun 1;11(4):e00311-23. doi: 10.1128/spectrum.00311-23 (PMC10433870; doi:10.1128/spectrum.00311-23)
Supplement: Supplemental file 2 — Table S1. Download spectrum.00311-23-s0002.docx, DOCX file, 0.02 MB [file spectrum.00311-23-s0002.docx]

Supplemental Table S1. Metadata for 71 MRSA strains isolated from human in 2018 of publicly available on NCBI.

| MRSA isolates | Host | BioProject | GenBank assembly accession | MLST | *SCCmec* Type | *spa* Type | Geographic region |
| --- | --- | --- | --- | --- | --- | --- | --- |
| 199 | Homo sapiens | PRJNA783074 | GCA_022494545.1 | ST88 | None | t17757 | China |
| 697 | Homo sapiens | PRJNA783074 | GCA_022494565.1 | ST59 | IV | t437 | China |
| GD-2 | Homo sapiens | PRJNA764055 | GCA_021328575.1 | ST5 | II | t2460 | China |
| GD-3 | Homo sapiens | PRJNA764055 | GCA_021328595.1 | ST239 | II | t037 | China |
| GD-4 | Homo sapiens | PRJNA764055 | GCA_021328615.1 | ST239 | III | t037 | China |
| GD-5 | Homo sapiens | PRJNA764055 | GCA_021328655.1 | ST5 | II | t002 | China |
| GD-6 | Homo sapiens | PRJNA764055 | GCA_021328635.1 | ST5 | II | Unknown | China |
| GD-7 | Homo sapiens | PRJNA764055 | GCA_021328675.1 | ST5 | II | t2460 | China |
| GD-8 | Homo sapiens | PRJNA764055 | GCA_021328695.1 | ST7611 | V | t4549 | China |
| GD-9 | Homo sapiens | PRJNA764055 | GCA_021328705.1 | ST5 | II | t002 | China |
| HCW | Homo sapiens | PRJNA493547 | GCA_004168825.1 | ST59 | V | t441 | China |
| MS4o8 | Homo sapiens | PRJNA511962 | GCA_003992475.1 | ST338 | V | t437 | China |
| NM-1 | Homo sapiens | PRJNA764055 | GCA_021327415.1 | ST5 | II | Unknown | China |
| NM-2 | Homo sapiens | PRJNA764055 | GCA_021327475.1 | ST5 | II | t2460 | China |
| NM-3 | Homo sapiens | PRJNA764055 | GCA_021327495.1 | ST5 | II | t2460 | China |
| NM-4 | Homo sapiens | PRJNA764055 | GCA_021327515.1 | ST5 | II | Unknown | China |
| NM-5 | Homo sapiens | PRJNA764055 | GCA_021327535.1 | ST5 | II | t2460 | China |
| NM-6 | Homo sapiens | PRJNA764055 | GCA_021327555.1 | ST5 | II | t2460 | China |
| NXNE | Homo sapiens | PRJNA658738 | GCA_015101795.1 | ST630 | None | t4549 | China |
| P11 | Homo sapiens | PRJNA493547 | GCA_004168725.1 | ST59 | V | t441 | China |
| P12 | Homo sapiens | PRJNA493547 | GCA_004168835.1 | ST59 | V | t441 | China |
| P13 | Homo sapiens | PRJNA493547 | GCA_004136935.1 | ST59 | V | t441 | China |
| P14 | Homo sapiens | PRJNA493547 | GCA_004168635.1 | ST59 | V | t441 | China |
| P15 | Homo sapiens | PRJNA493547 | GCA_004168815.1 | ST59 | V | t441 | China |
| RJ1267 | Homo sapiens | PRJNA598122 | GCA_009857055.1 | ST630 | V | t4549 | China |
| SA.1B | Homo sapiens | PRJNA505899 | GCA_009764615.1 | ST5 | II | t002 | China |
| SA17 | Homo sapiens | PRJNA561287 | GCA_008121315.1 | ST59 | IV | t437 | China |
| SAC4A | Homo sapiens | PRJNA577181 | GCA_009873845.1 | ST4513 | IV | t437 | China |
| SAC4B | Homo sapiens | PRJNA577181 | GCA_009873815.1 | ST4513 | IV | t437 | China |
| SAC5A | Homo sapiens | PRJNA577181 | GCA_009873735.1 | ST4513 | IV | t437 | China |
| SAC5B | Homo sapiens | PRJNA577181 | GCA_009874685.1 | ST4513 | IV | t437 | China |
| SAC5C | Homo sapiens | PRJNA577181 | GCA_009874635.1 | ST4513 | IV | t437 | China |
| SAW1 | Homo sapiens | PRJNA577395 | GCA_009914455.1 | ST59 | IV | t172 | China |
| SAW2 | Homo sapiens | PRJNA577181 | GCA_009873795.1 | ST59 | IV | t172 | China |
| SAW3 | Homo sapiens | PRJNA577181 | GCA_009873755.1 | ST59 | IV | t172 | China |
| SH-10 | Homo sapiens | PRJNA764055 | GCA_021327955.1 | ST7576 | II | t2460 | China |
| SH-11 | Homo sapiens | PRJNA764055 | GCA_021327975.1 | ST7576 | II | t2460 | China |
| SH-12 | Homo sapiens | PRJNA764055 | GCA_021328015.1 | ST630 | V | t2196 | China |
| SH-13 | Homo sapiens | PRJNA764055 | GCA_021328025.1 | ST7576 | II | t2460 | China |
| SH-14 | Homo sapiens | PRJNA764055 | GCA_021327995.1 | ST7576 | II | Unknown | China |
| SH-15 | Homo sapiens | PRJNA764055 | GCA_021328055.1 | ST7576 | II | t2460 | China |
| SH-7 | Homo sapiens | PRJNA764055 | GCA_021327875.1 | ST7576 | II | t2460 | China |
| SH-8 | Homo sapiens | PRJNA764055 | GCA_021327915.1 | ST5 | II | t5076 | China |
| SH-9 | Homo sapiens | PRJNA764055 | GCA_021327935.1 | ST5 | II | t264 | China |
| SKLX80731 | Homo sapiens | PRJNA769995 | GCA_021404005.1 | ST22 | None | t309 | China |
| SKLX83059 | Homo sapiens | PRJNA769995 | GCA_021403865.1 | ST22 | None | t309 | China |
| SKLX83971 | Homo sapiens | PRJNA735348 | GCA_018912865.1 | ST72 | IV | t324 | China |
| SKLX83977 | Homo sapiens | PRJNA735348 | GCA_018912775.1 | ST72 | IV | t324 | China |
| T22-2 | Homo sapiens | PRJNA587799 | GCA_012277165.1 | ST1 | IV | t114 | China |
| WH-1 | Homo sapiens | PRJNA764055 | GCA_021328135.1 | ST5 | II | t2460 | China |
| WH-10 | Homo sapiens | PRJNA764055 | GCA_021328315.1 | ST5 | II | t2460 | China |
| WH-11 | Homo sapiens | PRJNA764055 | GCA_021328335.1 | ST5 | II | t2460 | China |
| WH119 | Homo sapiens | PRJNA658738 | GCA_015101945.1 | ST630 | V | t4549 | China |
| WH-12 | Homo sapiens | PRJNA764055 | GCA_021328355.1 | ST5 | II | t9363 | China |
| WH-13 | Homo sapiens | PRJNA764055 | GCA_021328375.1 | ST5 | II | t2460 | China |
| WH-16 | Homo sapiens | PRJNA764055 | GCA_021328415.1 | ST5 | II | t2460 | China |
| WH-17 | Homo sapiens | PRJNA764055 | GCA_021328455.1 | ST630 | V | t4549 | China |
| WH-18 | Homo sapiens | PRJNA764055 | GCA_021328495.1 | ST5 | II | t4549 | China |
| WH-19 | Homo sapiens | PRJNA764055 | GCA_021328475.1 | ST5 | II | t2460 | China |
| WH-2 | Homo sapiens | PRJNA764055 | GCA_021328175.1 | ST5 | II | t2460 | China |
| WH-21 | Homo sapiens | PRJNA764055 | GCA_021328535.1 | ST5 | II | t2460 | China |
| WH-3 | Homo sapiens | PRJNA764055 | GCA_021328155.1 | ST5 | II | t2460 | China |
| WH39 | Homo sapiens | PRJNA657972 | GCA_016496025.1 | ST630 | V | t2196 | China |
| WH-4 | Homo sapiens | PRJNA764055 | GCA_021328195.1 | ST5 | II | t2460 | China |
| WH-5 | Homo sapiens | PRJNA764055 | GCA_021328215.1 | ST5 | II | t2460 | China |
| WH-6 | Homo sapiens | PRJNA764055 | GCA_021328235.1 | ST5 | II | t2460 | China |
| WH60 | Homo sapiens | PRJNA658738 | GCA_015101995.1 | ST630 | V | t4549 | China |
| WH-7 | Homo sapiens | PRJNA764055 | GCA_021328255.1 | ST5 | II | t2460 | China |
| WH-8 | Homo sapiens | PRJNA764055 | GCA_021328275.1 | ST5 | II | t2460 | China |
| WH-9 | Homo sapiens | PRJNA764055 | GCA_021328295.1 | ST239 | III | t030 | China |
| WH99 | Homo sapiens | PRJNA658738 | GCA_015101955.1 | ST630 | V | t4549 | China |
