# Supplementary material for: Molecular Surveillance of MRSA in Raw Milk Provides Insight into MRSA Cross Species Evolution
Source: Microbiol Spectr. 2023 Jun 1;11(4):e00311-23. doi: 10.1128/spectrum.00311-23 (PMC10433870; doi:10.1128/spectrum.00311-23)
Supplement: Supplemental file 3 — Table S2. Download spectrum.00311-23-s0003.docx, DOCX file, 0.03 MB [file spectrum.00311-23-s0003.docx]

Supplemental Table S2. Antimicrobial resistance profiles of LA-MRSA isolates.

| MRSA isolates | Antimicrobial resistance  profiles of MRSA | Antimicrobial agents | | | | | | | | | | | |
| --- | --- | --- | --- | --- | --- | --- | --- | --- | --- | --- | --- | --- | --- |
|  |  | PEN | OXA | FOX | GEN | SXT | SF | CC | ERY | OFX | VAN | DOX | LZD |
| GD9 | OXA-PEN-CC-ERY-SF-FOX | R | R | R | S | S | R | R | R | I | S | S | S |
| GD21 | OXA-PEN-CC-ERY-SF-FOX | R | R | R | S | S | R | R | R | S | S | S | S |
| GD22 | OXA-PEN-CC-ERY-SF-FOX | R | R | R | S | S | R | R | R | S | S | S | S |
| GD23 | OXA-PEN-CC-ERY-SF-FOX | R | R | R | S | S | R | R | R | S | S | S | S |
| GD24 | OXA-PEN-CC-ERY-SF-FOX | R | R | R | S | S | R | R | R | S | S | S | S |
| GD25 | OXA-PEN-CC-ERY-SF-FOX | R | R | R | S | S | R | R | R | S | S | S | S |
| GD26 | OXA-PEN-CC-ERY-SF-FOX | R | R | R | S | S | R | R | R | S | S | S | S |
| GD27 | OXA-PEN-CC-ERY-SF-FOX | R | R | R | S | S | R | R | R | S | S | S | S |
| GD28 | OXA-PEN-CC-ERY-SF-FOX | R | R | R | S | S | R | R | R | S | S | S | S |
| GD29 | OXA-PEN-CC-ERY-SF-FOX | R | R | R | S | S | R | R | R | S | S | S | S |
| GD30 | OXA-PEN-CC-ERY-SF-FOX | R | R | R | S | S | R | R | R | S | S | S | S |
| GD11 | OXA-PEN-CC-ERY-SF-FOX | R | R | R | S | S | R | R | R | S | S | S | S |
| GD12 | OXA-PEN-CC-ERY-SF-FOX | R | R | R | S | S | R | R | R | S | S | S | S |
| GD13 | OXA-PEN-CC-ERY-SF-FOX | R | R | R | S | S | R | R | R | S | S | S | S |
| GD14 | OXA-PEN-CC-ERY-SF-FOX | R | R | R | S | S | R | R | R | S | S | S | S |
| GD16 | OXA-PEN-CC-ERY-SF-FOX | R | R | R | S | S | R | R | R | S | S | S | S |
| GD17 | OXA-PEN-CC-ERY-SF-FOX | R | R | R | S | S | R | R | R | S | S | S | S |
| GD18 | OXA-PEN-CC-ERY-SF-FOX | R | R | R | S | S | R | R | R | I | S | S | S |
| GD19 | OXA-PEN-CC-ERY-SF-FOX | R | R | R | S | S | R | R | R | S | S | S | S |
| GD20 | OXA-PEN-CC-ERY-SF-FOX | R | R | R | S | S | R | R | R | S | S | S | S |
| GD10 | OXA-PEN-CC-ERY-SF-FOX | R | R | R | S | S | R | R | R | S | S | S | S |
| HN29 | GEN-PEN-CC-ERY-FOX | R | S | R | R | S | S | R | R | S | S | S | S |
| HN30 | GEN-PEN-CC-ERY-FOX | R | S | R | R | S | S | R | R | S | S | S | S |
| HN31 | OXA-PEN-SXT-CC-ERY-SF-FOX | R | R | R | I | R | R | R | R | S | S | S | S |
| ZJ52 | GEN-PEN-CC-ERY-FOX | R | S | R | R | S | S | R | R | R | S | S | S |
| ZJ57 | PEN-SXT | R | S | S | S | R | S | S | S | I | S | S | S |
| SC46 | GEN-PEN-SXT-CC-ERY-SF | R | S | S | R | R | R | R | R | I | S | S | S |
| SC48 | PEN-SF | R | S | S | S | S | R | S | S | S | S | S | S |
| SC52 | PEN-CC-ERY-SF | R | S | S | S | S | R | R | R | I | S | S | S |
| SC53 | OXA-PEN-CC-ERY-SF | R | R | S | S | S | R | R | R | S | S | S | S |
| SC54 | OXA-PEN-CC-ERY-SF | R | R | S | S | S | R | R | R | S | S | S | S |
| SC55 | OXA-PEN-CC-ERY-SF | R | R | S | S | S | R | R | R | S | S | S | S |
| SC56 | OXA-PEN-CC-ERY-SF | R | R | S | S | S | R | R | R | S | S | S | S |
| SC57 | OXA-PEN-CC-ERY-SF | R | R | S | S | S | R | R | R | S | S | S | S |
| SC58 | OXA-PEN-CC-ERY-SF | R | R | S | S | S | S | R | R | S | S | S | S |
| SC60 | OXA-PEN-CC-ERY-SF | R | R | S | S | S | R | R | R | S | S | S | S |
| SC61 | OXA-PEN-CC-ERY-SF | R | R | S | S | S | R | R | R | S | S | S | S |
| SC62 | OXA-PEN-CC-ERY-SF | R | R | S | S | S | R | R | R | S | S | S | S |
| SC63 | OXA-PEN-CC-ERY-SF | R | R | S | S | S | R | R | R | S | S | S | S |
| SH9 | PEN-ERY-SF-FOX | R | S | R | S | S | R | S | R | S | S | S | S |
| SH28 | OXA-PEN-SF-FOX | R | R | R | S | S | R | S | S | S | S | S | S |
| SH43 | PEN-ERY-SF-FOX | R | S | R | S | S | R | S | R | S | S | S | S |
| SH44 | OXA-PEN-CC-FOX | R | R | R | S | S | S | S | R | S | S | S | S |
| SH48 | PEN-ERY-SF-FOX | R | S | R | S | S | R | S | R | S | S | S | S |
| SH49 | OXA-PEN-CC-FOX | R | R | R | S | S | S | S | R | S | S | S | S |
| SH52 | PEN-CC-FOX | R | S | R | S | S | S | S | R | S | S | S | S |
| SH56 | GEN-OXA-PEN-SXT-CC-SF-FOX-OFX | R | R | R | R | R | R | R | S | R | S | S | S |
| SH65 | GEN-OXA-PEN-SXT-CC-SF-FOX-OFX | R | R | R | R | R | R | R | S | R | S | S | S |
| SH81 | GEN-OXA-PEN-CC-ERY-SF-FOX-OFX | R | R | R | R | S | R | R | R | R | S | S | S |

Note: PEN (Penicillin), OXA (Oxacillin), FOX (Cefoxitin), GEN (Gentamicin), SXT (Trimethoprim-sulfamethoxazole), SF (Sulfisoxazole), CC (Clindamycin), ERY (Erythromycin), OFX (Ofloxacin), VAN (Vancomycin), DOX (Doxycycline), LZD (Linezolid). R (resistant), I (intermediate) and S (susceptible).
